# On the Convergence and Stability Results for a New General Iterative Process

**DOI:** 10.1155/2014/852475

**Published:** 2014-09-02

**Authors:** Kadri Doğan, Vatan Karakaya

**Affiliations:** Department of Mathematical Engineering, Yildiz Technical University, Davutpasa Campus, Esenler, 34220 Istanbul, Turkey

## Abstract

We put forward a new general iterative process. We prove a convergence result as well as a stability result regarding this new iterative process for weak contraction operators.

## 1. Introduction and Preliminaries

Throughout this paper, by *N*, we denote the set of all positive integers. In this paper, we obtain results on the stability and strong convergence for a new iteration process (3) in an arbitrary Banach space by using weak contraction operator in the sense of Berinde [1]. Also, we obtain that the iteration procedure (3) can be useful method for solution of delay differential equations. To obtain solution of delay differential equation by using fixed point theory, some authors have done different studies. One can find these works in [2, 3]. Many results of stability have been established by some authors using different contractive mappings. The first study on the stability of the Picard iteration under Banach contraction condition was done by Ostrowski [4]. Some other remarkable results on the concept of stability can be found in works of the following authors involving Harder and Hicks [5, 6], Rhoades [7, 8], Osilike [9], Osilike and Udomene [10], and Singh and Prasad [11]. In 1988, Harder and Hicks [5] established applications of stability results to first order differential equations. Osilike and Udomene [10] developed a short proof of stability results for various fixed point iteration processes. Afterward, in following studies, same technique given in [10] has been used, by Berinde [12], Olatinwo [13], Imoru and Olatinwo [14], Karakaya et al. [15], and some authors.

Let (*E*, *d*) be complete metric space and *T* : *E* → *E* a self-map on *E*; and the set of fixed points of *T* in *E* is defined by *F*_*T*_ = {*p* ∈ *E* : *Tp* = *p*}. Let {*x*_*n*_}_*n*∈*N*_ ⊂ *E* be the sequence generated by an iteration involving *T* which is defined by

(1) $$\begin{array}{c} x_{n+1}=f\left(T,x_{n}\right)\;n=0,1,\ldots , \end{array}$$

where *x*_0_ ∈ *E* is the initial point and *f* is a proper function. Suppose that sequence {*x*_*n*_}_*n*∈*N*_ converges to a fixed point *p* of *T*. Let {*y*_*n*_}_*n*∈*N*_ ⊂ *E* and set

(2) $$\begin{array}{c} \epsilon_{n}=d\left(y_{n+1},f\left(T,y_{n}\right)\right)\;n=0,1,\ldots . \end{array}$$

Then, the iteration procedure (1) is said to be *T* stable or stable with respect to *T* if and only if lim⁡_*n*→*∞*_ *ϵ*_*n*_ = 0 implies lim⁡_*n*→*∞*_ *y*_*n*_ = *p*.

Now, let *C* be a convex subset of a normed space *E* and *T* : *C* → *C* a self-map on *E*. We introduce a new two-step iteration process which is a generalization of Ishikawa iteration process as follows:

(3) $$\begin{array}{c} x_{0}=x\in C,\\ f\left(T,x_{n}\right)=\left(1-{℘}_{n}\right)x_{n}+\xi_{n}Tx_{n}+\left({℘}_{n}-\xi_{n}\right)Ty_{n},\\ y_{n}=\left(1-\zeta_{n}\right)x_{n}+\zeta_{n}Tx_{n}, \end{array}$$

for *n* ≥ 0, where {*ξ*_*n*_},  {*℘*_*n*_}, and  {*ζ*_*n*_} satisfy the following conditions

(C_1_)
*℘*_*n*_ ≥ *ξ*_*n*_,
(C_2_)
{*℘*_*n*_−*ξ*_*n*_}_*n*=0_^*∞*^, {*℘*_*n*_}_*n*=0_^*∞*^, {*ζ*_*n*_}_*n*=0_^*∞*^, {*ξ*_*n*_}_*n*=0_^*∞*^ ∈ [0,1],
(C_3_)
∑_*n*=0_^*∞*^ *℘*_*n*_ = *∞*.

In the following remark, we show that the new iteration process is more general than the Ishikawa and Mann iteration processes.

**Remark 1 .**

1. If *ξ*_*n*_ = 0, then (3) reduces to the Ishikawa iteration process in [16].
2. If *ζ*_*n*_ = 0, then (3) reduces to the Mann iteration process in [17].

**Lemma 2 (see [18]).**

If *δ* is a real number such that 0 ≤ *δ* < 1 and {*ϵ*_*n*_}_*n*∈*N*_ is a sequence of positive real numbers such that lim⁡_*n*→*∞*_ *ϵ*_*n*_ = 0, then for any sequence of positive numbers {*u*_*n*_}_*n*∈*N*_ satisfying

(4) $$\begin{array}{c} u_{n+1}\leq \delta u_{n}+\epsilon_{n}\;n=0,1,\ldots \end{array}$$

one has

(5) $$\begin{array}{c} \underset{n\to \infty }{\lim}u_{n}=0. \end{array}$$

**Lemma 3 (see [2]).**

Let {*s*_*n*_}_*n*∈*N*_ be a sequence of positive real numbers including zero satisfying

(6) $$\begin{array}{c} s_{n+1}\leq \left(1-\mu_{n}\right)s_{n}. \end{array}$$

If {*μ*_*n*_} ⊂ (0,1) and ∑_*n*=0_^*∞*^ *μ*_*n*_ = *∞*, then lim⁡_*n*→*∞*_ *s*_*n*_ = 0.

A mapping *T* : *C* → *E* is said to be contraction if there is a fixed real number *a* ∈ [0,1) such that

(7) $$\begin{array}{c} ||Tx-Ty||\leq a||x-y|| \end{array}$$

for all *x*, *y* ∈ *C*.

This contraction condition has been generalized by many authors. For example, Kannan [19] shows that there exists *b* ∈ [0, 1/2) such that, for all *x*, *y* ∈ *C*,

(8) $$\begin{array}{c} ||Tx-Ty||\leq b\left[||x-Tx||+||y-Ty||\right]. \end{array}$$

Chatterjea [20] shows that there exists *c* ∈ [0, 1/2) such that, for all *x*, *y* ∈ *C*,

(9) $$\begin{array}{c} ||Tx-Ty||\leq c\left[||x-Ty||+||y-Tx||\right]. \end{array}$$

In 1972, Zamfirescu [21] obtained the following theorem.

**Theorem 4 (see [21]).**

Let (*X*, *d*) be a complete metric space and *T* : *X* → *X* a mapping for which there exist real numbers *a*, *b*, and *c* satisfying *a* ∈ (0,1),  *b*, *c* ∈ (0, 1/2) such that, for each pair *x*, *y* ∈ *X*, at least one of the following conditions is performed:

- *d*(*Tx*, *Ty*) ≤ *ad*(*x*, *y*),
- *d*(*Tx*, *Ty*) ≤ *b*[*d*(*x*, *Tx*) + *d*(*y*, *Ty*)],
- *d*(*Tx*, *Ty*) ≤ *c*[*d*(*x*, *Ty*) + *d*(*y*, *Tx*)].

Then *T* has a unique fixed point *p* and the Picard iteration {*x*_*n*_}_*n*∈*N*_ defined by

(10) $$\begin{array}{c} x_{n+1}=Tx_{n}\;n=0,1,2,\ldots \end{array}$$

converges to *p* for any arbitrary but fixed *x*_0_ ∈ *X*.

In 2004, Berinde introduced the definition which is a generalization of the above operators.

**Definition 5 (see [1]).**

A mapping *T* is said to be a weak contraction operator, if there exist *L* ≥ 0 and *δ* ∈ (0,1) such that

(11) $$\begin{array}{c} ||Tx-Ty||\leq \delta ||x-y||+L||x-Tx|| \end{array}$$

for all *x*, *y* ∈ *E*.

**Theorem 6 (see [1]).**

Let (*E*, ||·||) be Banach space. Assume that *C*⊆*E* is a nonempty closed convex subset and *T* : *C* → *C* is a mapping satisfying (11). Then *F*(*T*) ≠ *∅*.

**Definition 7 (see [22]).**

Let {*u*_*n*_}_*n*∈*N*_ and {*v*_*n*_}_*n*∈*N*_ be two iteration processes and let both {*u*_*n*_}_*n*∈*N*_ and {*v*_*n*_}_*n*∈*N*_ be converging to the same fixed point *p* of a self-mapping *T*. Assume that

(12) $$\begin{array}{c} \underset{n\to \infty }{\lim}\frac{||u_{n}-p||}{||v_{n}-p||}=0. \end{array}$$

Then, it is said that {*u*_*n*_}_*n*∈*N*_ converges faster than {*v*_*n*_}_*n*∈*N*_ to fixed point *p* of *T*.

The rate of convergence of the Picard and Mann iteration processes in terms of Zamfirescu operators in arbitrary Banach setting was compared by Berinde [22]. Using this class of operator, the Mann iteration method converges faster than the Ishikawa iteration method that was shown by Babu and Vara Prasad [23]. After a short time, Qing and Rhoades [24] showed that the claim of Babu and Vara Prasad [23] is false. There are many studies which have been made on the rate of convergence as given in [15, 25, 26] which are just a few of them.

## 2. Main Results

**Theorem 8 .**

Let *C* be a nonempty closed convex subset of an arbitrary Banach space *E* and let *T* : *C* → *C* be a mapping satisfying (11). Let {*x*_*n*_}_*n*∈*N*_ be defined through the new iteration (3) and *x*_0_ ∈ *E*, where {*℘*_*n*_−*ξ*_*n*_}_*n*=0_^*∞*^, {*℘*_*n*_}_*n*=0_^*∞*^{*ξ*_*n*_}, {*ζ*_*n*_} ∈ [0,1] with *℘*_*n*_ satisfying ∑_*n*=0_^*∞*^ *℘*_*n*_ = *∞*,  *℘*_*n*_ ≥ *ξ*_*n*_. Then {*x*_*n*_}_*n*∈*N*_ converges strongly to fixed point of *T*.

**Proof**

From Theorems 4 and 6, it is clear that *T* has a unique fixed point in *C* and *F*(*T*) ≠ *∅*.

From (3), we have

(13) ||xn+1−p|| =||(1−℘n)xn+(℘n−ξn)Tyn+ξnTxn−p|| ≤(1−℘n)||xn−p||+(℘n−ξn)||Tyn−p||+ξn||Txn−p|| ≤(1−℘n)||xn−p||+(℘n−ξn)δ||yn−p||  +(℘n−ξn)L||p−Tp||+ξnδ||xn−p||+ξnL||p−Tp|| =[1−℘n+ξnδ]||xn−p||+(℘n−ξn)δ||yn−p||  +℘nL||p−Tp||.

In addition,

(14) ||yn−p||=||(1−ζn)xn+ζnTxn−p||≤(1−ζn)||xn−p||+ζn||Txn−p||≤(1−ζn)||xn−p||+ζnδ||xn−p||+ζnL||p−Tp||=(1−ζn(1−δ))||xn−p||+ζnL||p−Tp||.

Substituting (14) in (13), we have the following estimates:

(15) ||xn+1−p|| ≤[1−℘n+ξnδ]||xn−p||  +(℘n−ξn)δ[(1−ζn(1−δ))||xn−p||+ζnL||p−Tp||]  +℘nL||p−Tp|| =[1−℘n+ξnδ+(℘n−ξn)δ(1−ζn(1−δ))]||xn−p||  +[(℘n−ξn)δζn+℘n]L||p−Tp||.

Since ||*p* − *Tp*|| = 0, we have

(16) ||xn+1−p||≤[1−℘n(1−δ)]||xn−p||||xn+1−p||≤(1−℘n(1−δ))||xn−p||||xn−p||≤(1−℘n−1(1−δ))||xn−1−p||||xn−1−p||≤(1−℘n−2(1−δ))||xn−2−p||⋮||x2−p||≤(1−℘1(1−δ))||x1−p||||x1−p||≤(1−℘0(1−δ))||x0−p||||xn+1−p||≤∏i=0n[1−℘i(1−δ)]×||x0−p||≤||x0−p||×e(∑i=0n[−℘i(1−δ)])=||x0−p||×e(−(1−δ)∑i=0n℘i)

for all *n* ∈ *N*.

Since 0 < *δ* < 1,  *℘*_*n*_ ∈ [0,1], and ∑_*n*=0_^*∞*^ *℘*_*n*_ = *∞*, we have

(17) lim⁡n→∞sup⁡||xn+1−p|| ≤lim⁡n→∞sup⁡||x0−p||×e(−(1−δ)∑i=0n℘i)≤0.

So lim⁡_*n*→*∞*_||*x*_*n*_ − *p*|| = 0 yields *x*_*n*_ → *p* ∈ *F*(*T*). This completes the proof of theorem.

**Theorem 9 .**

Let (*E*, ||·||) be Banach space and *T* : *E* → *E* a self-mapping with fixed point *p* with respect to weak contraction condition in the sense of Berinde (11). Let {*x*_*n*_}_*n*∈*N*_ be iteration process (3) converging to fixed point of *T*, where *℘*_*n*_ ≥ *ξ*_*n*_ and {*℘*_*n*_−*ξ*_*n*_}_*n*=0_^*∞*^, {*℘*_*n*_}_*n*=0_^*∞*^, {*ξ*_*n*_}_*n*=0_^*∞*^, {*ζ*_*n*_}_*n*=0_^*∞*^ ∈ [0.1] such that 0 < *℘* ≤ *℘*_*n*_  for all *n*. Then two-step iteration process is *T* stable.

**Proof**

Let {*x*_*n*_}_*n*∈*N*_ be iteration process (3) converging to *p*. Assume that {*y*_*n*_}_*n*∈*N*_ ⊂ *E* is an arbitrary sequence in *E*. Set

(18) ϵn=||yn+1−(1−℘n)yn+(℘n−ξn)Tvn+ξnTyn||n=0,1,…,

where *v*_*n*_ = (1 − *ζ*_*n*_)*y*_*n*_ + *ζ*_*n*_ *Ty*_*n*_. Suppose that lim⁡_*n*→*∞*_ *ϵ*_*n*_ = 0. Then, we shall prove that lim⁡_*n*→*∞*_ *y*_*n*_ = *p*. Using contraction condition (11), we have

(19) ||yn+1−p||≤||yn+1−(1−℘n)yn+(℘n−ξn)Tvn+ξnTyn|| +||(1−℘n)yn+(℘n−ξn)Tvn+ξnTyn−p||≤εn+||(1−℘n)yn+(℘n−ξn)Tvn+ξnTyn−p||≤εn+(1−℘n)||yn−p||+(℘n−ξn)||Tvn−p|| +ξn||Tyn−p||≤εn+(1−℘n)||yn−p||+(℘n−ξn)δ||vn−p|| +ξnδ||yn−p||+(℘n−ξn)L||p−Tp|| +ξnL||p−Tp||=εn+(1−℘n+ξnδ)||yn−p|| +(℘n−ξn)δ||vn−p|| +(℘n−ξn)L||p−Tp||+ξnL||p−Tp||.

We estimate ||*v*_*n*_ − *p*|| in (19) as follows:

(20) ||vn−p||=||(1−ζn)yn+ζnTyn−p||≤(1−ζn)||yn−p||+ζn||Tyn−p||≤(1−ζn)||yn−p||+ζnδ||yn−p|| +ζnφ(||p−Tp||)=(1−ζn(1−δ))||yn−p||+ζnφ(||p−Tp||).

Substituting (20) in (19), we have

(21) ||yn+1−p|| ≤εn+(1−℘n+ξnδ)||yn−p||+℘nL||p−Tp||  +(℘n−ξn)δ[(1−ζn(1−δ))||yn−p||+ζnL||p−Tp||] =εn+[1−℘n+ξnδ+(℘n−ξn)δ(1−ζn(1−δ))]  ×||yn−p||+[(℘n−ξn)ζnδ+℘n]L||p−Tp||.

Since ||*p* − *Tp*|| = 0, we have

(22) ||yn+1−p|| ≤εn+[1−℘n+ξnδ+(℘n−ξn)δ(1−ζn(1−δ))]  ×||yn−p||≤εn+[1−℘n(1−δ)]||yn−p||.

Since 0 < 1 − *℘*_*n*_(1 − *δ*) < 1 and using Lemma 2, we obtain lim⁡_*n*→*∞*_ *y*_*n*_ = *p*.

Conversely, letting lim⁡_*n*→*∞*_ *y*_*n*_ = *p*, we show that lim⁡_*n*→*∞*_ *ε*_*n*_ = 0 as follows:

(23) εn=||yn+1−(1−℘n)yn−(℘n−ξn)Tvn−ξnTyn||≤||yn+1−p||+||p−(1−℘n)yn−(℘n−ξn)Tvn−ξnTyn||≤||yn+1−p||+(1−℘n)||yn−p||+(℘n−ξn)||Tvn−p|| +ξn||Tyn−p||≤||yn+1−p||+[1−℘n+δξn]||yn−p|| +(℘n−ξn)δ||vn−p||≤||yn+1−p||+[1−℘n+δξn] ×||yn−p||+(℘n−ξn)δ(1−ζn(1−δ))||yn−p||≤||yn+1−p||+[1−℘n+δξn+(℘n−ξn)δ(1−ζn(1−δ))]||yn−p||≤||yn+1−p||+[1−℘n(1−δ)]||yn−p||.

Since lim⁡_*n*→*∞*_||*y*_*n*_ − *p*|| = 0, it follows that lim⁡_*n*→*∞*_ *ε*_*n*_ = 0. Therefore the iteration scheme is *T* stable.

**Example 10 (see [24]).**

Let *T* : [0,1] → [0,1], *Tx* = *x*/2, *℘*_*n*_, *ξ*_*n*_, *ζ*_*n*_, *ϑ*_*n*_ = 0, *n* = 1,2,…, 15, and $\xi_{n}=1/2-2/\sqrt{n}$,  ${℘}_{n}=1/2+2/\sqrt{n}$, $\zeta_{n}=\vartheta_{n}=4/\sqrt{n}$, for all *n* ≥ 16.  It is easy to show that *T* is a weak contraction operator satisfying (11) with a unique fixed point 0. Furthermore, for all *n* ≥ 16, $4/\sqrt{n},1/2-2/\sqrt{n},1/2+2/\sqrt{n}\in \left[0,1\right]$, and $\sum_{n=0}^{\infty }\left(1/2+2/\sqrt{n}\right)=\infty$. Then the new iterative process is faster than the Ishikawa iterative process. Assume that *u*_0_ = *w*_0_ ≠ 0 is initial point for the new and Ishikawa iterative processes, respectively. Firstly, we consider the new iterative process, and we have

(24) un+1=(1−℘n)un+(℘n−ξn)T((1−ζn)un+ζnTun) +ξnTun=(1−(12+2n))un +((12+2n)−(12−2n)) ×T((1−4n)un+4nTun) +(12−2n)Tun=(1−(12+2n))un +((12+2n)−(12−2n)) ×12((1−4n)un+4n12un) +(12−2n)12un=(12−2n)un +2n(1−2n)un+(14−1n)un=(12−2n+2n−4n+14−1n)un=(1−1n−4n−14)un=∏i=16n(1−1i−4i−14)u0.

Secondly, we consider the Ishikawa iterative process, and we have

(25) wn+1=(1−ζn)wn+ζnT((1−ϑn)wn+ϑnTwn)=(1−4n)wn+4nT((1−4n)wn+4nTwn)=(1−4n)wn+4n12((1−4n)wn+4n12wn)=(1−4n)wn+2n((1−4n)wn+2nwn)=(1−4n)wn+2n(1−2n)wn=(1−4n+2n−4n)wn=(1−2n−4n)wn=∏i=16n(1−2i−4i)w0.

Now, taking the above two equalities, we obtain

(26) |un+1−0wn+1−0| =|∏i=16n(1−1/i−4/i−1/4)u0∏i=16n(1−2/i−4/i)w0| =|∏i=16n(1−1/i−4/i−1/4)(1−2/i−4/i)| =|∏i=16n[1−−1/i+1/41−2/i−4/i]| =|∏i=16n[1−i−4i4i−8i−16]|.

It is clear that

(27) $$\begin{array}{c} 0\leq \underset{n\to \infty }{\lim}\overset{n}{\underset{i=16}{\prod }}\left[1-\frac{i-4\sqrt{i}}{4i-8\sqrt{i}-16}\right]=0. \end{array}$$

Therefore, the proof is completed.

**Figure 1:**
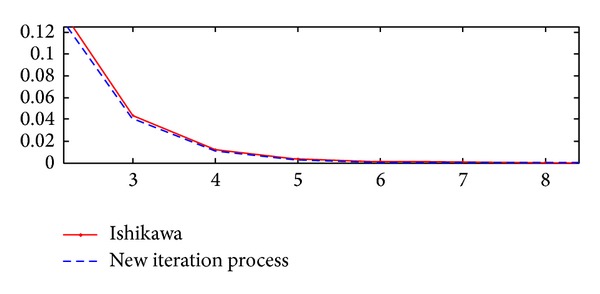
It shows the value functions found by successive steps of the Ishikawa and new iteration methods.

**Figure 2:**
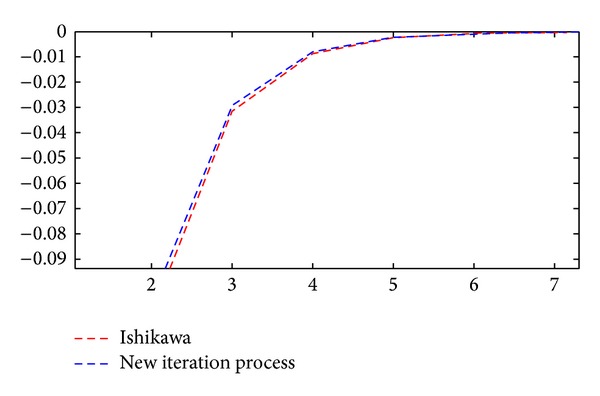
It shows the derivative functions of the Ishikawa and new iteration methods.

Now, we can give Table 1 and Figures 1 and 2 to support and reinforce our claim in the Example 10.

Finally, we check that this iteration procedure can be applied to find the solution of delay differential equations.

### 2.1. An Application

Throughout the rest of this paper, the space *C*[*a*, *b*] equipped with Chebyshev norm ||*x*−*y*||_*∞*_ = max⁡_*t*∈[*a*,*b*]_|*x*(*t*) − *y*(*t*)| denotes the space of all continuous functions. It is well known that *C*[*a*, *b*] is a real Banach space with respect to ||·||_*∞*_ norm; more details can be found in [2, 27].

Now, we will consider a delay differential equation such that

(28) $$\begin{array}{c} x^{\prime }\left(t\right)=g\left(t,x\left(t\right),x\left(t-\varsigma \right)\right),\;t\in \left[t_{0n},b\right] \end{array}$$

and an assumed solution

(29) $$\begin{array}{c} x\left(t\right)=\varphi \left(t\right),\;t\in \left[t_{0}-\varsigma ,t_{0}\right]. \end{array}$$

Assume that the following conditions are satisfied:

(C_1_)
*t*_0_, *b* ∈ *R*,  *ς* ≥ 0,
(C_2_)
*g* ∈ *C*([*t*_0_, *b*] × *R* ^2^, *R*),
(C_3_)
*φ* ∈ *C*([*t*_0_ − *ς*, *t*_0_], *R*),
(C_4_)
there exists the following inequality:
  (30) |g(t,γ1,γ2)−g(t,λ1,λ2)| ≤Kg[|γ1−λ1|+|γ2−λ2|]+L|γ1−Tγ1|,
  for all *γ*_*i*_, *λ*_*i*_ ∈ *R*  (*i* = 1,2) and *t* ∈ [*t*_0_, *b*] such that *K*_*g*_ > 0,
(C_5_)
2*K*_*g*_(*b* − *t*_0_) < 1, and according to a solution of problem (28)-(29) we infer the function *x* ∈ *C*([*t*_0_ − *ς*, *b*], *R*)∩*C* ^1^([*t*_0_, *b*], *R*). The problem can be reconstituted as follows:
(C_6_)
(31) x(t)={φ(t),if  t∈[t0−ς,t0],φ(t0)+∫t0tg(t,x(s),x(s−ς))ds,if  t∈[t0,b].
  Also, the map *T* : *C*([*t*_0_ − *ς*, *b*], *R*) → *C*([*t*_0_ − *ς*, *b*], *R*) is defined by the following form:
  (32) T(x)(t)={φ(t),if  t∈[t0−ς,t0],φ(t0)+∫t0tg(t,x(s),x(s−ς))ds,if  t∈[t0,b].

Using weak-contraction mapping, we obtain the following.

**Theorem 11 .**

We suppose that conditions (*C*_1_)–(*C*_5_) are performed. Then the problem (28)-(29) has a unique solution in *C*([*t*_0_ − *ς*, *b*], *R*)∩*C* ^1^([*t*_0_, *b*], *R*).

**Proof**

We consider iterative process (3) for the mapping *T*. The fixed point of *T* is shown via *p* such that *Tp* = *p*.

For the first part, that is, for *t* ∈ [*t*_0_ − *ς*, *t*_0_], it is clear that lim⁡_*n*→*∞*_ *x*_*n*_ = *p*. Therefore, letting *t* ∈ [*t*_0_, *b*], we obtain

(33) ||xn+1−p||∞ =||(1−℘n)xn+ξnTxn+(℘n−ξn)Tyn−p||∞ ≤(1−℘n)||xn−p||∞+ξn||Txn−Tp||∞  +(℘n−ξn)||Tyn−Tp||∞ ≤(1−℘n)||xn−p||∞ +ξn||Txn−Tp||∞+(℘n−ξn)||Tyn−Tp||∞ =(1−℘n)||xn−p||∞+ξnmax⁡t∈[t0−ς,b]|Txn(t)−Tp(t)|  +(℘n−ξn)max⁡t∈[t0−ς,b]|Tyn(t)−Tp(t)| =(1−℘n)||xn−p||∞  +ξnmax⁡t∈[t0−ς,b]|φ(t0)+∫t0tg(t,xn(s),xn(s−ς))ds−φ(t0)−∫t0tg(t,p(s),p(s−ς))ds|  +(℘n−ξn)max⁡t∈[t0−ς,b]|φ(t0) +∫t0tg(t,yn(s),yn(s−ς))ds −φ(t0)−∫t0tg(t,p(s),p(s−ς))ds| =(1−℘n)||xn−p||∞  +ξnmax⁡t∈[t0−ς,b]|∫t0tg(t,xn(s),xn(s−ς))ds−∫t0tg(t,p(s),p(s−ς))ds|  +(℘n−ξn)max⁡t∈[t0−ς,b]|∫t0tg(t,yn(s),yn(s−ς))ds−∫t0tg(t,p(s),p(s−ς))ds| ≤(1−℘n)||xn−p||∞+ξn  ×max⁡t∈[t0−ς,b]∫t0t|g(t,xn(s),xn(s−ς))−g(t,p(s),p(s−ς))|ds  +(℘n−ξn)max⁡t∈[t0−ς,b]∫t0t|g(t,yn(s),yn(s−ς))−g(t,p(s),p(s−ς))|ds ≤(1−℘n)||xn−p||∞  +ξnmax⁡t∈[t0−ς,b]∫t0t[Kg(|xn(s)−p(s)| +|xn(s−ς)−p(s−ς)|) +L|xn(s)−Txn(s)|]ds +(℘n−ξn)max⁡t∈[t0−ς,b]∫t0t[Kg(|yn(s)−p(s)|+|yn(s−ς)−p(s−ς)|)+L|yn(s)−Tyn(s)|]ds.

Hence, we obtain

(34) ||xn+1−p||∞≤(1−℘n)||xn−p||∞ +ξnKg(b−t0)max⁡t∈[t0−ς,b]{|xn(t)−p(t)|+|xn(t)−p(t)|} +ξnL(b−t0)max⁡t∈[t0−ς,b]|xn(t)−Txn(t)| +(℘n−ξn)Kg(b−t0) ×max⁡t∈[t0−ς,b]{|yn(t)−p(t)|+|yn(t)−p(t)|} +(℘n−ξn)L(b−t0) ×max⁡t∈[t0−ς,b]|yn(t)−Tyn(t)|=(1−℘n)||xn−p||∞+2ξnKg(b−t0) ×max⁡t∈[t0−ς,b]|xn(t)−p(t)| +2(℘n−ξn)Kg(b−t0) ×max⁡t∈[t0−ς,b]|yn(t)−p(t)|=(1−℘n)||xn−p||∞ +2ξnKg(b−t0)||xn−p||∞ +2(℘n−ξn)Kg(b−t0)||yn−p||∞=(1−℘n+2ξnKg(b−t0))||xn−p||∞ +2(℘n−ξn)Kg(b−t0)||yn−p||∞.

By continuing this way, we have

(35) ||yn−p||∞ =||(1−ζn)xn+ζnTxn−p||∞ ≤(1−ζn)||xn−p||∞+ζn||Txn−p||∞ ≤(1−ζn)||xn−p||∞ +ζnmax⁡t∈[t0−ς,b]|Txn(t)−Tp(t)| =(1−ζn)||xn−p||∞ +ζnmax⁡t∈[t0−ς,b]|φ(t0)+∫t0tg(t,xn(s),xn(s−ς))ds−φ(t0)−∫t0tg(t,p(s),p(s−ς))ds| =(1−ζn)||xn−p||∞ +ζnmax⁡t∈[t0−ς,b]|∫t0tg(t,xn(s),xn(s−ς))ds−∫t0tg(t,p(s),p(s−ς))ds| ≤(1−ζn)||xn−p||∞ +ζnmax⁡t∈[t0−ς,b]∫t0t|g(t,xn(s),xn(s−ς))−g(t,p(s),p(s−ς))|ds ≤(1−ζn)||xn−p||∞ +ζn∫t0t[Kg(|xn(s)−p(s)|+|xn(s−ς)−p(s−ς)|)+L|xn(s)−Txn(s)|]ds.

Hence, we obtain

(36) ||yn−p||∞ ≤(1−ζn)||xn−p||∞  +ζnKg(b−t0)max⁡t∈[t0−ς,b]{|xn(t)−p(t)|+|xn(t−ς)−p(t−ς)|}  +ζnL(b−t0)max⁡t∈[t0−ς,b]|xn(t)−Txn(t)| ≤(1−ζn)||xn−p||∞  +ζn2Kg(b−t0)max⁡t∈[t0−ς,b]|xn(t)−p(t)| =(1−ζn)||xn−p||∞+ζn2Kg(b−t0)||xn−p||∞ =(1−ζn(1−2Kg(b−t0)))||xn−p||∞.

Substituting (36) into (34), we obtain

(37) ||xn+1−p||∞≤(1−℘n+2ξnKg(b−t0))||xn−p||∞ +(℘n−ξn)2Kg(b−t0) ×(1−ζn(1−2Kg(b−t0)))||xn−p||∞=(1−℘n+2ξnKg(b−t0)+(℘n−ξn)2Kg(b−t0)×(1−ζn(1−2Kg(b−t0))))||xn−p||∞.

Since (1 − 2*K*_*g*_(*b* − *t*_0_)) < 1, we have

(38) $$\begin{array}{c} {||x_{n+1}-p||}_{\infty }\leq \left(1-\zeta_{n}\left(1-2K_{g}\left(b-t_{0}\right)\right)\right){||x_{n}-p||}_{\infty }. \end{array}$$

We take *ζ*_*n*_(1 − 2*K*_*g*_(*b* − *t*_0_)) = *μ*_*n*_ < 1 and ||*x*_*n*_−*p*||_*∞*_ = *s*_*n*_, and then the conditions of Lemma 3 immediately imply lim⁡_*n*→*∞*_||*x*_*n*_−*p*||_*∞*_ = 0.

## Figures and Tables

**Table 1:** Iterative values of the rate of convergence to zero of the Ishikawa and new iteration process.

*x*_*n*_	Ishikawa	New iteration process
*x*_1_	1,990000000000000	1,990000000000000
*x*_2_	0,556472918237748	0,541618812060050
*x*_3_	0,155609099865344	0,147412531445900
*x*_4_	0,043513693420310	0,040121306615323
*x*_5_	0,012167935658745	0,010919826345371
*x*_6_	0,003402576213543	0,002972051946272
*x*_7_	0,000951478148280	0,000808904142975
*x*_8_	0,000266066242117	0,000220159648738
*x*_9_	0,000074401335777	0,000059920908248
*x*_10_	0,000020805190171	0,000016308689016
*x*_11_	0,000005817851703	0,000004438740086
*x*_12_	0,000001626872822	0,000001208093031
*x*_13_	0,000000454929983	0,000000328806991
*x*_14_	0,000000127214179	0,000000089491483
*x*_15_	0,000000035573490	0,000000024356920
*x*_16_	0,000000009947580	0,000000006629229
*x*_17_	0,000000002781688	0,000000001804279
*x*_18_	0,000000000777856	0,000000000491071
*x*_19_	0,000000000217516	0,000000000133655
*x*_20_	0,000000000060825	0,000000000036377
*x*_21_	0,000000000017009	0,000000000009901
*x*_22_	0,000000000004756	0,000000000002695
*x*_23_	0,000000000001330	0,000000000000733
*x*_24_	0,000000000000372	0,000000000000200
*x*_25_	0,000000000000104	0,000000000000054
*x*_26_	0,000000000000029	0,000000000000015
*x*_27_	0,000000000000008	0,000000000000004
*x*_28_	0,000000000000002	0,000000000000001
*x*_29_	0,000000000000001	0,000000000000000
